# Programmable multiphoton quantum interference in a single spatial mode

**DOI:** 10.1126/sciadv.adj0993

**Published:** 2024-04-19

**Authors:** Lorenzo Carosini, Virginia Oddi, Francesco Giorgino, Lena M. Hansen, Benoit Seron, Simone Piacentini, Tobias Guggemos, Iris Agresti, Juan C. Loredo, Philip Walther

**Affiliations:** ^1^ University of Vienna, Faculty of Physics,Vienna Center for Quantum Science and Technology (VCQ), 1090 Vienna, Austria.; ^2^ Christian Doppler Laboratory for Photonic Quantum Computer, Faculty of Physics, University of Vienna, 1090 Vienna, Austria.; ^3^ Dipartimento di Fisica, Politecnico di Milano, Piazza Leonardo da Vinci, 32, I-20133 Milano, Italy.; ^4^ Quantum Information and Communication, Ecole polytechnique de Bruxelles, CP 165/59, Université libre de Bruxelles (ULB), 1050 Brussels, Belgium.; ^5^ Istituto di Fotonica e Nanotecnologie, Consiglio Nazionale delle Ricerche (IFN-CNR), Piazza Leonardo da Vinci, 32, I-20133 Milano, Italy.; ^6^ Remote Sensing Technology Institute, German Aerospace Center DLR, Münchener Straße 20, 82234 Weßling, Germany.; ^7^ University of Vienna, Research Network for Quantum Aspects of Space Time (TURIS), Boltzmanngasse 5, 1090 Vienna, Austria.; ^8^ Institute for Quantum Optics and Quantum Information (IQOQI) Vienna, Austrian Academy of Sciences, Boltzmanngasse 3, 1090 Vienna, Austria.

## Abstract

The interference of nonclassical states of light enables quantum-enhanced applications reaching from metrology to computation. Most commonly, the polarization or spatial location of single photons are used as addressable degrees of freedom for turning these applications into praxis. However, the scale-up for the processing of a large number of photons of these architectures is very resource-demanding due to the rapidly increasing number of components, such as optical elements, photon sources, and detectors. Here, we demonstrate a resource-efficient architecture for multiphoton processing based on time-bin encoding in a single spatial mode. We use an efficient quantum dot single-photon source and a fast programmable time-bin interferometer to observe the interference of up to eight photons in 16 modes, all recorded only with one detector, thus considerably reducing the physical overhead previously needed for achieving equivalent tasks. Our results can form the basis for a future universal photonics quantum processor operating in a single spatial mode.

## Teaser

Innovative architecture leverages time-bin encoding for resource-efficient multiphoton processing in quantum computing.

## INTRODUCTION

Multiphoton interference lies at the heart of many optical quantum technologies. An optical quantum computer (*1*, *2*) itself is in essence a large photonic multimode interferometer producing outcomes that cannot be efficiently obtained by an otherwise classical device. Here, typically, one starts by preparing as many single photons as possible in different spatial modes, which are then fed into bulk-based (*3*) or integrated (*4*, *5*) interferometric networks. The most complex multiphoton experiments thus far have prepared sources of tens of single photons (*6*, *7*), as well as squeezed states of light (*8*, *9*), interfering in circuits with more than 100 modes and detectors (*10*). While these are impressive achievements, it is clear that approaches of this kind are incredibly resource-demanding, simply requiring the control of unfeasibly many elements simultaneously—from large numbers of phase-locked sources to high-voltage active electro-optical components and costly superconducting nanowire single-photon detectors (SNSPDs), to name a few. It is thus essential to develop methods that can still produce equivalent nonclassical statistics but efficiently use the available physical resources.

The most advanced technologies for producing multiphoton sources to date are either based on probabilistic frequency–conversion processes in nonlinear crystals (*11*, *12*) or obtained deterministically from the spontaneous emission of atomic transitions (*13*–*16*) and subsequent time-space demultiplexing (*3*, *17*). The former can be run either below a pump threshold to produce heralded single photons, but with efficiencies kept low to mitigate the effect of unwanted higher-order photon emission, or run above threshold to produce squeezed states instead, however requiring complex phase-locking systems. On the other hand, the latter technology can deterministically produce single photons that are also efficiently collected, with state-of-the-art (fiber) source efficiencies beyond 50%. Starting from one such source, active demultiplexing enables the construction of multiphoton sources. However, this demultiplexing step may not even be necessary. A standard one-photon source already contains all the necessary single photons—they are all in different temporal modes, only sharing the same spatial trajectory. Thus, should one have access to devices that can alter their time-bin photon statistics, multiphoton interference can entirely occur in one single spatial mode.

For photonic qubits, time-domain architectures have been proposed (*18*) using looped geometries for optical paths and repeated use of the same optical components. Related experiments, involving up to four photons, have been reported (*19*–*21*). Recently, these concepts have also been extended to the continuous-variable framework, yielding notable results (*22*–*24*). This approach allowed achieving a quantum computational advantage with a programmable Gaussian boson sampler (*25*), making use of three fiber loops to synthesize a multimode-entangled Gaussian state and subsequent time-space demultiplexing and sampling using an array of detectors. However, continuous-variable implementations encounter notable limitations, particularly regarding their high sensitivity to losses, which degrades the quality of the quantum state. In contrast, discrete-variable encoding emerges as an appealing alternative, with loss-tolerant schemes capable of producing large entangled quantum states via heralded quantum gate operations (*26*).

Here, we demonstrate resource-efficient interference and discrete-variable processing with a considerable number of particles, carried out with a very limited number of physical resources: one single-photon source, one programmable loop interferometer, and one single-photon detector. That is, by combining a quantum dot (QD)–based photon source, from which we measure single-photon count rates at 17.1 MHz, together with a low-loss fast reconfigurable optical processor and one highly efficient SNSPD, we observe the interference of up to 8 photons in 16 modes, where all the multiphoton processing is carried out by analyzing the time tags of a single detector. Extensions of our results can enable a future resource-efficient universal quantum photonics processor.

## RESULTS

**Fig. 1.**
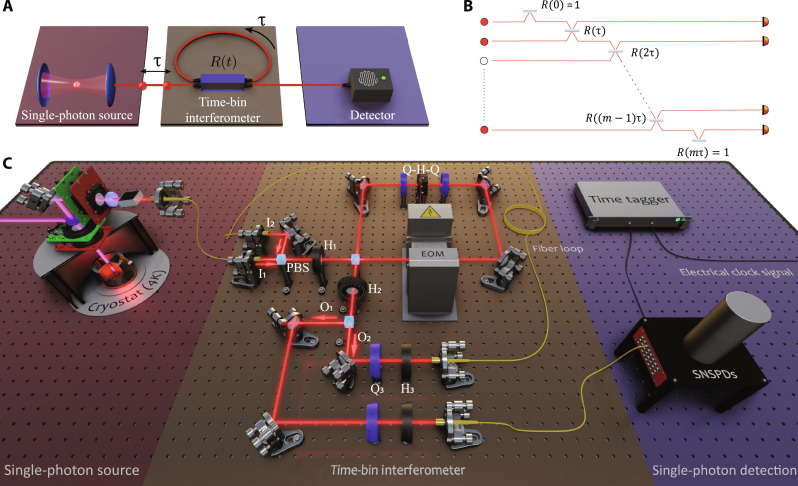
Time-bin multiphoton interferometric network. (**A**) Time-bin multiphoton processor. One single-photon source, one time-bin loop interferometer, and one detector implement the processor. (**B**) Representation of time-bin multimode interferometer. The combination of a tunable beam splitter and delay loop implements a network of *m* modes: an arbitrary sequence of beam splitter operations between consecutive time bins, with *m* − 1 reflectivities *R*(*k*τ), *k* = 1, …, *m* − 1. The input time bins contain either vacuum or a single photon. Boundary initial and final reflectivities *R*(0) = *R*(*m*τ) = 1 correctly initialize and terminate the time-bin experiment. (**C**) Depiction of the setup. The source (left) is an InGaAs QD coupled to a micropillar cavity and kept at 4 K inside a cryostat. A confocal setup is used to pump and collect resonance fluorescence and then sent to a time-bin interferometer: an effective time-varying beam splitter with two inputs I_1_ and I_2_ and two outputs O_1_ and O_2_, with one output connected (looped) to one input via a 100-ns fiber-based delay (~20 m). The free-space electro-opic phase modulator (EOM) controls the time-varying reflectivity, which can be reconfigured to any value every 100 ns. HWPs H_1_ and H_2_ are kept at π/8°. H_3_ and QWPs Q_3_ ensure that light traversing the loop arrives with vertical polarization into loop input I_2_ again. After traversing the loop a number of times, all photons and time bins exit the interferometer and are detected with only one detector. The resulting statistics is reconstructed by postprocessing events registered by the time tagger.

Figure 1 describes the architecture we follow. It uses a time-bin interferometer composed of active and tunable linear optical elements and looped spatial trajectories, as proposed in (*18*) and further studied in (*27*, *28*). The first step consists in triggering a single-photon source at time intervals τ to prepare a train of *n* single photons in *m* designed time bins along a single spatial trajectory. The following step propagates the photon stream through a time-bin multimode interferometer, the core of which consists of a beam splitter with time-varying reflectivity. Here, one output of the beam splitter is connected back (looped) to one of its inputs and traverses a delay matched to the arrival of a subsequent input photon after the time τ. In this way, the device implements an arbitrary beam splitter action between consecutive time bins. This loop-based architecture is equivalent to a network with *m* modes that are pairwise connected via beam splitters with time-programmable reflectivities, as illustrated in Fig. 1B. We want to emphasize that this time bin–based scheme gives access to a universal linear optics network when adding just a second phase–stable loop (*24*, *29*).

One main experimental challenge here is finding a physical implementation of an active looped interferometer such that it is fast-reconfigurable, low-loss, and capable of modifying and measuring the time-bin statistics entirely in a single spatial mode. Note that previous implementations of loop-based architectures have not yet met these conditions simultaneously. We go beyond previous experiments and demonstrate that these conditions can be fulfilled (see Fig. 1C). Our time-varying beam splitter is built from a polarizing beam splitter (PBS)–based Sagnac interferometer containing a free-space electro-optical phase modulator (phase-EOM; from QUBIG GmbH) with a phase that can be reconfigured to an arbitrary value [−π, π] every 100 ns (*30*, *31*). The PBSs used here have throughput losses below 1% and polarization extinction of more than 2000:1 for both output ports. The loss of the (antireflection-coated) phase-EOM is also below 1%. This allows building a fast-reconfigurable (10 MHz), high-visibility (0.998), and low-loss interferometer acting as our time-programmable beam splitter. The output of this device is looped to one of its inputs and undergoes an optical delay of 100 ns, such that two propagating time bins are always simultaneously arriving to the input ports of the tunable beam splitter. The fiber-based delay has an optical transmission of 94%, mainly originating from the nonunity fiber-coupling efficiency.

Our single-photon source consists of resonance fluorescence signal from a QD-cavity device in a sample acquired commercially from Quandela. Laser pulses with 80-MHz repetition rate coherently drive a trion transition—effectively, a two-level system at zero magnetic field. Our home-built optical setup is optimized to minimize collection losses, with a measured combined transmission of all free-space optical components of 0.89 ± 0.01. The single-photon free-space spatial mode is coupled to a single-mode fiber with a coupling efficiency of 0.93 ± 0.01, resulting in a first-lens to fiber polarized transmission of 0.83 ± 0.01. By driving our source at π-pulse excitation, we directly measure 17.1 MHz of single photons with a Single Quantum Eos SNSPD of 0.85 ± 0.02 efficiency. Under these conditions, we observe simultaneous high single-photon purity 1 − *g*^(2)^(0) = (98.61 ± 0.01)% and indistinguishability *I* = (94.21 ± 0.07)% (see Materials and Methods).

To run our experiment, we chop pulses from the pump laser using a fibered electro-optical amplitude modulator, which, upon excitation of the QD, prepares a train of *n* single photons in *m* ≥ *n* time bins separated by τ = 100 ns. As a result, each time bin is either occupied, or not, by a single photon. After traversing the looped interferometer, the output photonic time-bin modes are sent into an SNSPD connected to a time correlator—Time Tagger X, from Swabian Instruments—so that the output time-bin statistics can be revealed.

**Fig. 2.**
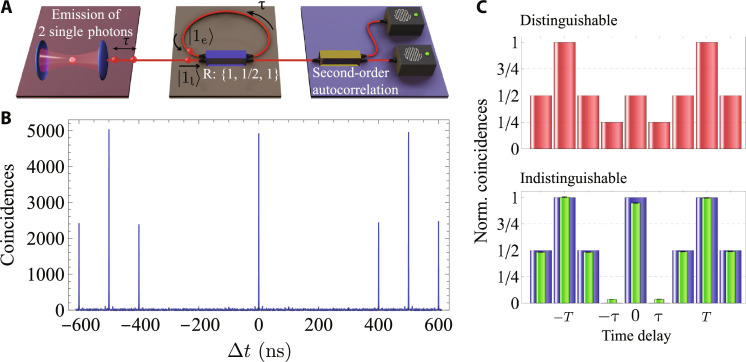
Time-bin HOM interference. (**A**) Protocol sequence. The source sends an early ∣1_e_〉 and late ∣1_l_〉 single-photon toward the time-varying looped interferometer. A sequence of reflectivities *R* : {1,1/2,1} implements the time-bin HOM protocol. The output is analyzed with a time-static beam splitter and two detectors. (**B**) Resulting second-order autocorrelation histogram. A series of coincidence peaks appear at different time delays. The signature of the time-bin HOM effect is the lack of coincidences at a delay equal to the photons’ initial temporal separation τ = 100 ns. (**C**) Normalized coincidences. Calculated for distinguishable photon input (red), indistinguishable photon input (blue), and extracted from our experimental data (green) by integrating the corresponding peak areas in a 3-ns window. The small error bars are estimated following Poissonian statistics of the detected events.

As a first test of our device, we performed a time-bin version of a Hong-Ou-Mandel (HOM) experiment (*32*). Here, we prepare our source such that two photons in consecutive time bins, early and late, are sent to the loop interferometer. The active beam splitter reflectivity, tuned via the phase of the EOM, is set to *R*(0) = 1 at the arrival of the first photon; hence, it is sent deterministically into the loop. After τ = 100 ns, the reflectivity is set to *R*(τ) = 1/2, so that the first and second photons interfere, traversing two inputs of a balanced beam splitter (see Fig. 2A). According to the rules that govern bosonic bunching, the two particles should leave the beam splitter along the same output port, either escaping the loop or, with equal probability, both coupled into the loop again. After another 100 ns, the reflectivity is programmed to *R*(2τ) = 1, such that the time bin within the loop exits entirely the setup and travels toward the detector. At the output of the time-bin interferometer, photon statistics is contained within one spatial mode, where in the ideal case, both photons bunch at one of two consecutive time bins, while terms with one photon in each time bin are suppressed. Note that the time bin before the first photon, as well as the one after the second photon, must be empty for the correct operation of the protocol.

To help illustrate the result of this case, only for this measurement, we use two SNSPDs and perform a standard second-order autocorrelation measurement at the time-bin interferometer output. Figure 2B displays the resulting coincidence histogram and contains (i) strong correlations at Δ*t* = 0, originating from the photon bunching terms—two photons occupying the same temporal mode lead to coincidences at zero delay after passing through a time-static beam splitter—and (ii) suppressed correlations at *Δt* = 100 ns—i.e., between consecutive time bins—which is a signature of the time-bin HOM effect. We evaluate the time-bin two-photon interference visibility via *V*^(2)^ = 1 − 2*C*_∣τ∣_/(*C*_∣τ∣_ + *C*_0_), where *C*_∣τ∣_ = *C*τ + *C*_−τ_ is the sum of areas of correlated peaks at *Δt* = ± τ and *C*_0_ is the area of the correlated peak at *Δt* = 0. Normalization implies *Cτ* + *C*_−τ_ + *C*_0_ = *C*, with *C* being the area of uncorrelated s at delays larger than the bin separation, located at *Δt* = T = ± 500 ns in Fig. 2B, with *T* being the period at which the experiment (sequence) is repeated. Note that for distinguishable particles, one expects *C*_τ_ = *C*_−τ_ = *C*/4 and *C*_0_ = *C*/2, whereas fully indistinguishable photons lead to *Cτ* = *C*_−τ_ = 0 and *C*_0_ = *C*. Figure 2C displays these values of correlated and uncorrelated peaks (coincidences) normalized to the reference *C*, calculated for the case of fully distinguishable particles (red bars) and the fully indistinguishable case (blue bars), together with the values obtained from our experiment (green bars). From our data, we obtain *V*^(2)^ = (85.97 ± 0.06)%. Note that this value is affected by several factors, such as residual multiphoton emission from the QD, photon distinguishability at increasing timescales (*33*, *34*), imperfect active switching of the time-bin interferometer, and imperfect modulation of the laser pump stream that allocates either a single photon or vacuum inside a time bin (see the Supplementary Materials for a more in-depth discussion of these factors).

One strong advantage of active time-bin interference is that the size of the implemented protocol can be increased and programmed without increasing the physical size of the experimental apparatus. For instance, the number of photons and modes is defined by choosing a pattern of time bins that contain, or not, a single-photon, and the specific linear-optical network is set by specifying time-varying values of reflectivities. Hence, this protocol allows to straightforwardly increase the number of interfering photons *n*, where the practical limit is set by overall experimental efficiencies leading to an exponentially decreasing rate of *n-*photon events.

**Fig. 3.**
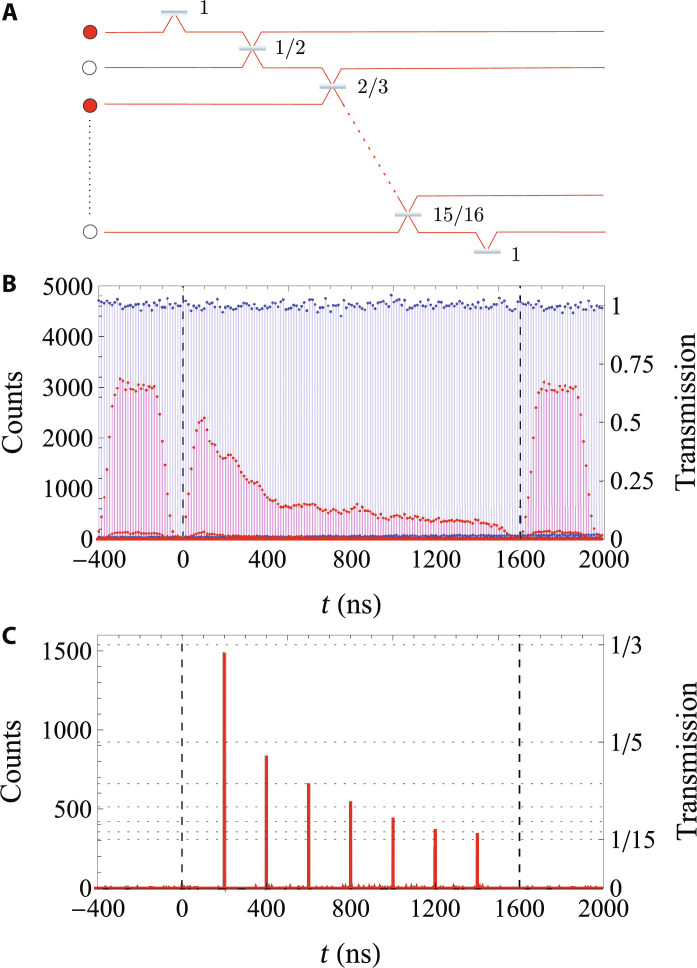
Time-varying network. (**A**) Eight-photon network. *n* = 8 single photons occupy odd positions in *m* = 16 modes, in a network with reflectivities *R_k_* = *k*/(*k* + 1), *k* = 1, ...,15. (**B**) Measured time-dependent counts at the output of the time-bin interferometer. The loop is blocked; hence, only the transmitted intensity is observed without (blue) and with (red) phase modulation. The area within the vertical dashed lines denotes the time lapse of interest, i.e., that containing 16 time bins separated by 100 ns. The time duration outside this window can be chosen to take any value, in consequence defining the effective repetition rate of the experiment. Modulation of the laser pump (pulse chopping) is off; thus, a peak appears every 12.5 ns. All time-varying values of transmission 1 − *R_k_* occur between *t* = 0 and *t* = 1.6 μs. (**C**) Pulse chopping on. Only eight photons can now appear among all time bins. Note that the first photon at *t* = 0 is not visible, as transmission at this point is zero [boundary reflectivity *R*(0) = 1 sends the photon into the loop]. This sequence of photons, synchronized with the sequence of reflectivities, performs the time-bin eight-photon protocol.

Accordingly, we now program our device to allow for the interference of a larger number of photons. In particular, we choose to place *n* photons in *m* = 2*n* modes, for *n* up to 8 single photons—number constrained mainly by our source efficiency. In every experiment, the first input time bin is deterministically routed into the loop, and the *m* + 1 input time bin is deterministically routed out from the loop. When the first and second time bins interfere, some amplitude escapes the loop, defining the first output time bin. The sequentially traveling photons keep interfering through the loop via the programmed beam splitter operations, and then after a time *m* × τ, the last beam splitter reflectivity is chosen such that the remaining amplitude in the loop deterministically leaves it, defining the *m*th output time bin. To showcase the programmability of our time-bin processor, we targeted specific sets of reflectivities; in particular, we choose *R_k_* = *k*/(*k* + 1) for *k* = 1, …, *m* − 1, motivated by their application in, e.g., investigating the quantum central limit theorem (*35*). Figure 3 shows one example of these time-varying reflectivities and network for an experiment with *n* = 8 photons in *m* = 16 modes. The reflectivity values are programmed by setting the voltage waveform produced by the arbitrary function generator that drives the phase-EOM. Note that in some cases, e.g., in boson sampling protocols, a number of modes scaling as *m* ~ *n*^2^ is preferred. In our case, our scaling choice suffices as we no longer benefit from further increasing the number of modes given the specifics of the implemented network.

**Fig. 4.**
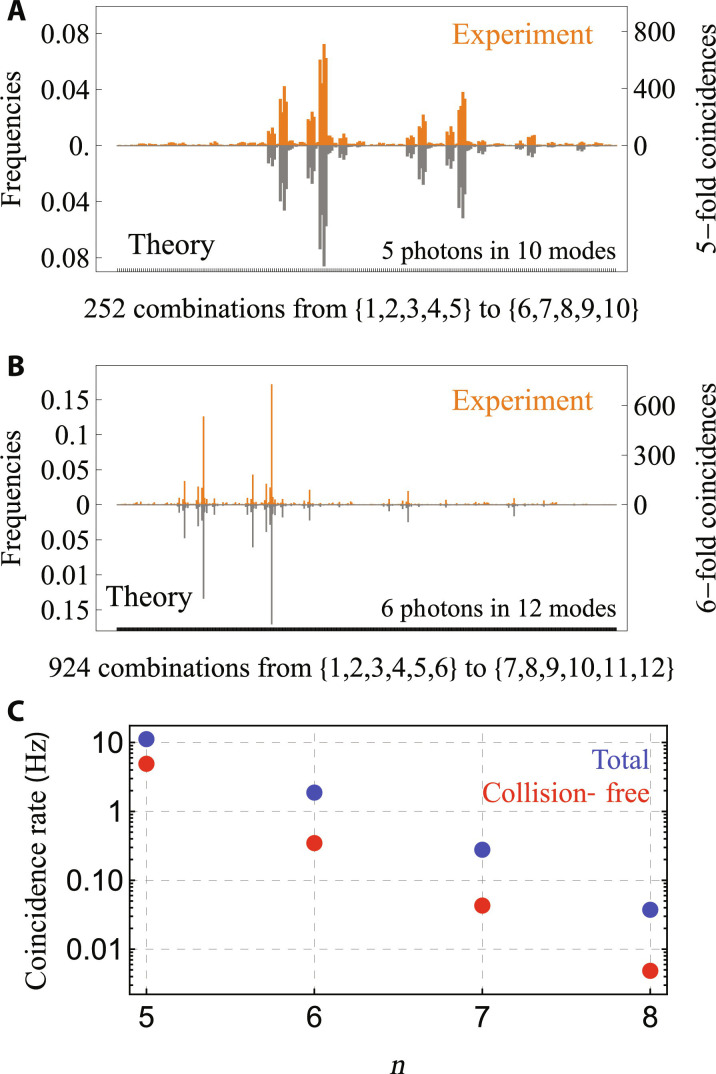
Reconstructed multiphoton output distribution. Experiment (orange) and theory (gray) output frequencies for (**A**) *n* = 5 photons in *m* = 10 modes, with reflectivities *R_k_* = (0.5, 0.6, 0.7, 0.8, 0.8, 0.8, 0.7, 0.5, 0.4), and photons occupying even input modes {2,4,6,8,10}; and for (**B**) *n* = 6 photons in *m* = 12 modes, with reflectivities *R_k_* = *k*/(*k* + 1), *k* = 1, ...,11, and photons in odd inputs {1,3,5,7,9,11}. (**C**) Coincidence rates. The measured rates (red dots) are only on the collision-free subspace. The total rates (blue dots) are estimated from dividing the measured ones by the accumulated probability of the collision-free subspace.

With these tools, we program multiphoton interference experiments with various numbers of photons. For example, Fig. 4 displays instances with 5 and 6 photons and distinct patterns of reflectivities. For each *n*-photon experiment, we sample the output time-bin distribution by measuring collision-free *n*-photon events, i.e., all those events in which *n* photons output the interferometer in *n* different output time-bin modes. The number of possible such events is given by $\left(\begin{array}{c} m\\ n \end{array}\right)$; by recording the amount of detected events for each of these combinations, we experimentally assess their relative frequencies, that is, output probabilities normalized within the collision-free subspace. These measurements are carried out using only one SNSPD and analyzing the registered time tags (see the Supplementary Materials). Compared to the spatial encoding approach, this feature circumvents the need for as many detectors as there are output modes, thus considerably reducing the physical overhead required, e.g., in quantum computing tasks. To obtain the expected output probabilities, we model the involved optical circuits, including the effect of optical losses, with software available both in Python (*36*, *37*) and in Julia (*38*). The agreement between experiment and theory is quantified by the statistical fidelity $F=\sum_{i}\sqrt{p_{i}^{\text{exp}}p_{i}^{\text{th}}}$ between their normalized distributions or frequencies. We find *F*^(5)^ = 0.939 ± 0.006 and *F*^(6)^ = 0.914 ± 0.009 for the cases with *n* = 5 and *n* = 6 photons, respectively. For *n* = 7 and *n* = 8 photons, it is no longer possible to collect the full output distribution, as in the smaller experiments, due to a restricted number of collected events compared to the total number of possible output collision-free configurations. Figure 4C displays the measured coincidence rates of our *n*-photon interference experiments. The rate of the collision-free events for 8 interfering photons is already at the 5-mHz level, at which point we stop adding particles. To provide further evidence of bosonic quantum interference in our interferometer, we adopt two validation techniques against alternative explanations for our experimental statistics, which are widely used for validating the boson sampling protocols on photonic platforms (*39*) (see the Supplementary Materials for further details on simulations and validation techniques).

**Fig. 5.**
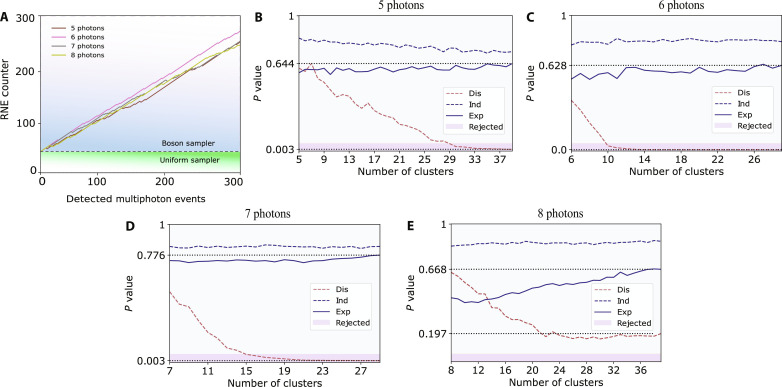
Experimental validation. (**A**) Validation against uniform sampler. For every detected multiphoton event, a row-norm estimator (RNE) is calculated, and a counter is updated according to its value. The increasing counter indicates that data are obtained from genuine bosonic interference. For clarity, we included only the first 300 randomly chosen events for each experiment. (**B** to **E**) Validation against distinguishable sampler for the experiments with 5, 6, 7, and 8 photons, respectively. The plots show the *P* values obtained through the machine learning–based validation method used to exclude that photons in our apparatus were not displaying bosonic interference. The dashed curves refer to numerically generated samples, which were used to select the hyperparameter values (namely, number of clusters and sample size) ensuring that the algorithm could effectively discard samples drawn from the distinguishable photon inputs and recognize as compatible those drawn from the indistinguishable photon inputs. The size of the compared samples ranges from 300 (for the eight-photon experiment) to 700 (for the six-photon experiment) events. Whenever the size of the experimental samples was larger than the chosen sample size, we randomly extracted samples from the available experimental data 100 times and evaluated the *P* value corresponding to the mean of the χ^2^ variables. Only for the case of *n* = 8, the small sample size is too low to conclusively reject samples drawn from the distinguishable photon input distribution. However, we can observe that the experimental *P* value converges closer to the indistinguishable sample case.

We first exclude the hypothesis that the observed distribution arises from the uniform scattering of the samples (*40*). Figure 5A depicts the results of this method for validating experiments with up to eight-photon interference. The increasing positive counters with registered multiphoton events indicate that our data do not result from a uniform sampler.

Furthermore, we apply a machine learning–based validation method (*41*) to rule out the hypothesis that our data originate from distinguishable photon input, hence one that displays no quantum interference. This technique is based on the comparison of two samples (a bona fide and a test one) and estimates the probability that they are drawn from the same probability distribution. Figure 5B shows the results of this method for the validation of our experiments with up to eight-photon interference. We take samples from the theoretical distributions derived from indistinguishable photon inputs (compatible to the bona fide samples), as well as others drawn from the alternative hypothesis of distinguishable particle inputs (to be rejected). The results of the simulated protocols are displayed as dashed curves in Fig. 5B, following their expected behavior. Notably, the experimental data (solid curves) closely follow the behavior of indistinguishable photon input, hence supporting the hypothesis for bosonic quantum interference.

## DISCUSSION

We experimentally demonstrated time-bin reconfigurable multiphoton quantum interference occurring within one spatial mode. The size of the implemented experiments was chosen and increased, while maintaining a constant number of (programmable) physical resources: one single-photon source, one active time-bin processor, and one detector. We used validation protocols to certify that our data originates from quantum interference, particularly with a recently introduced machine learning–based approach (*41*).

In practice, the size and complexity of the experiment are constrained mainly by source efficiencies, in our case, allowing the observation up to 8-photon interference. Note that when using only one loop in a time-bin experiment, the resulting collision-free *n*-photon rate also decreases for larger number of photons. This can be avoided by adding a second outer loop, as in (*18*, *24*), to allow for full connectivity in the optical circuit, in which case most *n*-photon events land in the collision-free subspace. Moreover, allowing for lost photons (*7*, *42*) is straightforward in our implementation and then increasing the rates of detected multiphoton events. However, in our current implementation, we restrain from this approach, as it might rapidly lead to classical output distributions.

Our results show that the architecture is highly resource-efficient in comparison to the standard spatial encoding approach, as it does not require active demultiplexing of a single-photon source or building arrays of multiple identical emitters. The feasibility of our architecture is underlined by observing tunable multiphoton interference for, in principle, arbitrary number of particles and modes. We expect that future developments will aim to add a second outer phase–stable loop for achieving universal linear optics quantum processing in a single spatial mode.

## MATERIALS AND METHODS

### Single-photon source

**Fig. 6.**
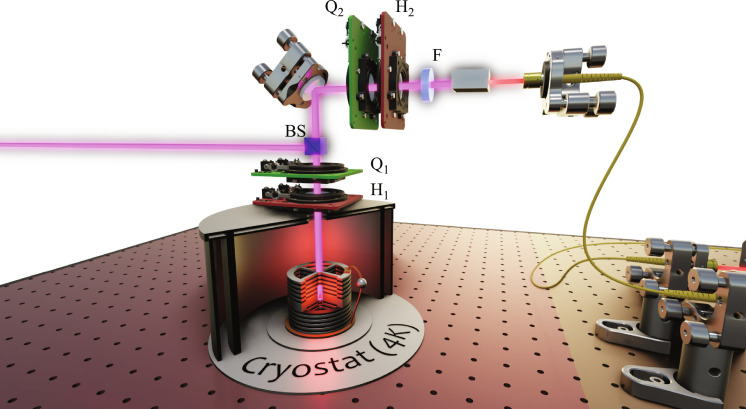
Source setup. BS, beam splitter.

**Fig. 7.**
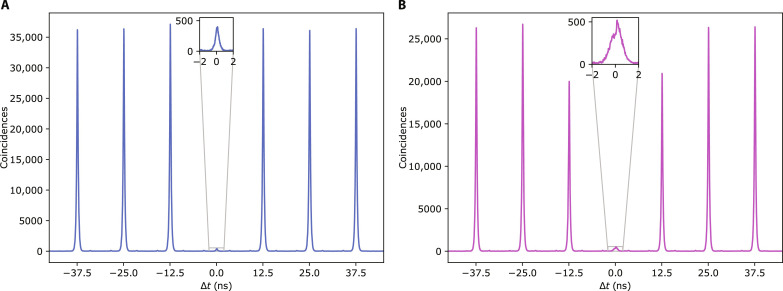
Source characterization. (**A**) Second-order autocorrelation measurement. We obtain a single-photon purity 1 − *g*^(2)^(0) = (98.61 ± 0.01)%. (**B**) Two-photon interference autocorrelation. The resulting photon indistinguishability is *I* = (94.21 ± 0.07)%. These values are obtained by integrating peak areas in a 3-ns window.

The single-photon emitter is a charged exciton of an InGaAs QD embedded in an electrically connected micropillar cavity. The sample is kept at ~4 K in a low-vibration closed-cycle Attocube cryostat—an Attodry800 system. The QD is pumped resonantly with a pulsed laser of 80-MHz repetition rate, spectrally shaped with a home-built 4-f system, to set a bandwidth of ~100 pm and match the QD wavelength of 922.3 nm. A confocal microscope setup (see Fig. 6) is used to both excite the QD and collect its resonance fluorescence. We place a 97:3 (transmission/reflection) beam splitter and use the reflection mode for excitation and the transmission mode for collection. A standard crossed-polarization scheme—made of two quarter–wave plates (QWPs) Q_1_ and Q_2_ and two half–wave plates (HWPs) H_1_ and H_2_—separates the laser pump from the single-photon signal. We place a 70-pm etalon filter (F) to remove the residual phonon-sideband emission, while leaving the zero-phonon line nearly unaffected. The single photons are then coupled to a single-mode fiber. When the collection polarization is set parallel to that of the excitation laser, we measure a transmission of all the collection path, from the first lens inside the cryostat to the output of the single-mode fiber, of 0.83 ± 001. At π-pulse excitation, we measure 17.1 MHz of high-quality single photons with an 85% efficient detection system. Figure 7 displays measurements of the single-photon purity and indistinguishability under these conditions.

### Time-bin interferometer

**Fig. 8.**
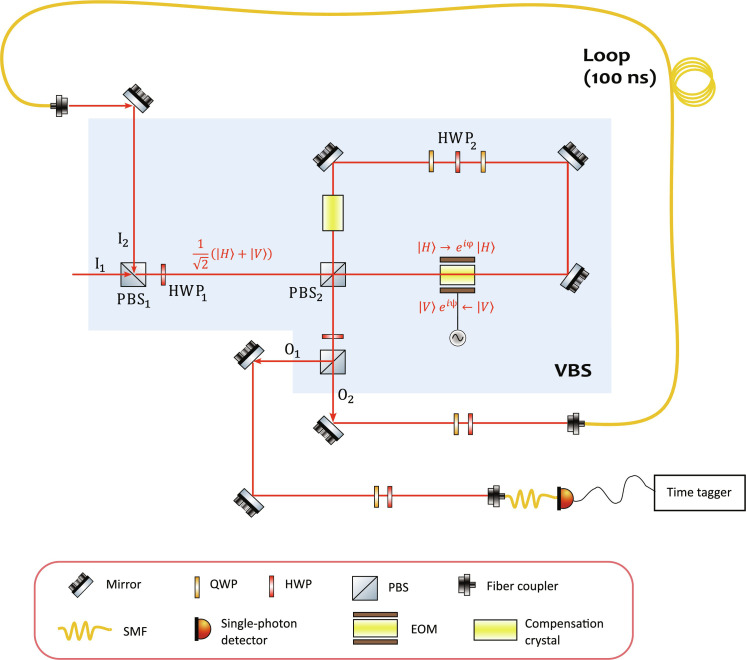
Sketch of the time-bin interferometer. VBS highlighted by shaded area. See the text for details.

**Fig. 9.**
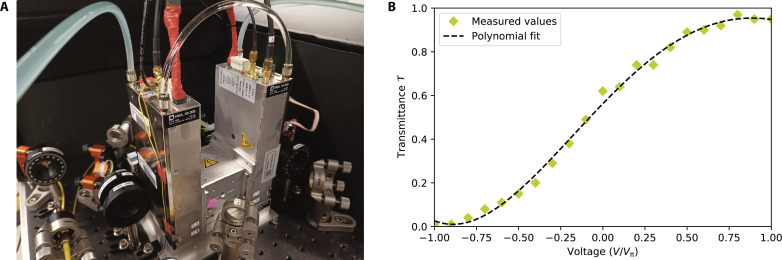
The electro-optic phase-EOM used in our experiment. (**A**) Picture of the device. (**B**) Measured transmittance of the VBS as a function of the voltage applied to the electro-optic crystal. Here, *V*π is the half-wave voltage of the crystal, i.e., the voltage required for inducing a phase change of π.

Trains of single photons from the source are directed toward the programmable time-bin interferometer, consisting of a variable beam splitter (VBS) and a fiber delay line of τ = 100 ns. The part of the setup implementing the VBS is highlighted by the shaded area in Fig. 8. Its input ports I_1_ and I_2_ are also the input ports of PBS_1_, while its output ports O_1_ and O_2_ coincide with the output ports of PBS_3_. Its core is a free-space PBS-based Sagnac interferometer, whose optical paths pass through the EOM (see picture in Fig. 9A). This phase-EOM is a 3-mm by 3-mm lithium niobate crystal embedded in a high-voltage (±300 V) amplifier manufactured by QUBIG GmbH. The AR-coated crystal has an optical transmittance of >99% at 930 nm and is mounted on a five-axis alignment stage that allows precision angle and height adjustments with respect to the laser beam. This device allows fast-phase modulation to an arbitrary value [−π, π], where a new value of phase can be set every 100 ns, thus corresponding to a repetition rate of up to 10 MHz.

In this setup, PBS_1_ and HWP_1_ prepare the state $|+\rangle =\frac{1}{\sqrt{2}}\left(|H\rangle +|V\rangle \right)$, where ∣*H*〉 and ∣*V*〉 are horizontal and vertical polarization states, respectively. The two components are then separated by the central PBS_2_ and counterpropagate in the Sagnac interferometer, passing through the EOM from opposite directions. Because of the birefringence of the crystal, the phase change induced by the EOM is different for the ∣*H*〉 and ∣*V*〉 components, such that when they recombine in PBS_2_, there is an induced phase shift between them. This leads to a variable transmittance *T* ∈ [0,1] on PBS_3_ as a function of the applied voltage (see Fig. 9B). Moreover, a motorized HWP_2_ sandwiched between two QWPs in the Sagnac interferometer allows setting a phase offset to calibrate half-wave voltages of the EOM and corresponding transmittance of PBS_3_. Note that the birefringent crystal of the EOM introduces a temporal walk-off between the ∣*H*〉 and ∣*V*〉 components, which is compensated with another identical, orthogonally oriented, crystal inside the Sagnac interferometer.

To run the experiments, an arbitrary function generator drives the EOM through a sequence of voltage levels every τ = 100 ns, thus introducing a different local phase shift on every time-bin mode. In this way, the device implements an arbitrary beam splitter action between consecutive time bins. The photons reflected by the tunable beam splitter (that is, leaving PBS_3_ from O_2_) are coupled into a ≈20-m single-mode optical fiber (SMF) and looped back to I_2_. Through this delay line, they undergo an optical delay of 100 ns, matched to the arrival of subsequent input photons. The fiber-based delay has an optical transmission of 94%, mainly originating from the nonunity fiber-coupling efficiency. Note that the photons coming from the fiber loop enter in the VBS from input I_2_ in the ∣*V*〉 state, so that after HWP_1_, their polarization state $|-\rangle =\frac{1}{\sqrt{2}}\left(|H\rangle -|V\rangle \right)$ is orthogonal to that of the photons entering the VBS from input port I_1_. Consequently, they experience complementary transmittances.

## Supplementary Materials

### This PDF file includes:

Supplementary Text Figs. S1 to S5 References
