# Supplementary material for: Programmable multiphoton quantum interference in a single spatial mode
Source: Sci Adv. 2024 Apr 19;10(16):eadj0993. doi: 10.1126/sciadv.adj0993 (PMC12697571; doi:10.1126/sciadv.adj0993)
Supplement: Supplementary file 1 — Supplementary Text Figs. S1 to S5 References [file sciadv.adj0993_sm.pdf]

Science Advances
AAAS

Supplementary Materials for

Programmable multiphoton quantum interference in a single spatial mode

Lorenzo Carosini *et al.*

Corresponding author: Lorenzo Carosini, lorenzo.carosini@univie.ac.at; Juan C. Loredo, juan.loredo@univie.ac.at

Sci. Adv. **10**, eadj0993 (2024)
DOI: 10.1126/sciadv.adj0993

This PDF file includes:

Supplementary Text
Figs. S1 to S5
References

1 Time-bin detection

As explained in the main text, our device acts by altering the time-bin statistics of the photons traversing through it. In each experimental run, we prepare a train of n photons in $m{>}n$ time-bins, equally separated by $\tau{=}100$ ns (each time-bin hosting one or zero photons). After interfering in the loop, they leave the experimental apparatus redistributed into configurations at the m output time-bins (*20*). Therefore, the key ingredient to sample their time-bin output distribution is the ability to uniquely recognize the output modes and measure the distribution of photons among them. In this work, we achieve this time-bin detection by using only one superconducting nanowire single-photon detector (SNSPD), and analyzing the timestamps of the detected events registered by a time-to-digital converter, Time-Tagger X from Swabian Instruments. The Time-Tagger Application Programmer's Interface (API) provides methods to control the hardware and to create measurements that are hooked onto the time-tag stream. Not only the API includes a set of standard measurements that cover common tasks relevant to photon counting, but it also allowed us to implement our own custom measurement.

Fig. S1 illustrates how our measurement works for a case with $n{=}3$ and $m{=}6$. The time tagger registers timestamps of single-photon events on a channel we call the "detector channel". At the same time, we send an electrical square pulse signal to another channel of the time tagger, whose period defines the duration of a single experimental period, thus acting as a "clock". At a later point, the raw time-tags are read from the resulting file to perform the desired analysis.

The first step is to synchronize the timestamps of the clock square pulses with the first time-bin we wish to examine. This can be done easily by inspecting at the correlation histogram between the clock and detector channels, see Fig. S2-a. In fact, this histogram clearly shows count peaks at the time bins where the photons leave the interferometer. The synchronization can then be performed by delaying (i.e., introducing a time delay on all the timestamps registered on) the clock channel, so that the first peak of the correlation histogram occurs at zero time difference, see Fig. S2-b.

With the timestamps of the two channels properly synchronized, for each experimental period marked by the clock, m time bins of 5 ns width are defined: the first coinciding with the clock timestamp, the second after a 100 ns delay, the third after a 200 ns delay, etc., see Fig. S1. Once the time bins have been identified, our program loops over all the timestamps and for each clock pulse examines whether there are single-photon detection timestamps in the corresponding time bins and, if so, in which ones. Thanks to this analysis we are able to determine, for each experimental period, into which time-bins n output photons landed.

The main limitation of our detection system is that the detector used does not photon-number resolve. Due to this constraint, we sampled the output time-bin distribution by measuring only collision-free n-photon events, i.e, all those events in which n photons leave the interferometer in n different output time-bin modes.

2 Simulations

We simulate our experiment as a chain of $m{-}1$ beamsplitters acting on m modes. We include measured 15% losses for each loop, originating from non-unity fibre-coupling efficiency, and imperfect mode-matching to the output of the time-bin interferometer. To include losses, we modify the number of simulated modes to $2m$ and indicate $1, ..., m$ as logical modes m_{log} and $m+1, ..., 2m$ as loss modes m_{loss}.

As an example, let us consider the 5 photons in 10 modes, with input state $|1010101010\rangle$, and we wish to calculate the probability of all output states that detect 5 photons, out of a total of $\binom{10}{5} = 252$ combinations. In the case of loss, the optical circuit will have 5 photons in 20

modes, of which $m_{log} = (1, ..., 10)$ model the setup and and $m_{loss} = (11, ..., 20)$ model the losses. Between each logical beamsplitter, we add a beamsplitter with reflectivity 85% with input/output modes $i, i+10$, see Fig. S3. Hence, the input state changes to $|1010101010\rangle \otimes |0\rangle^{\otimes 10}$, and the output states change accordingly. Doing so, the calculated probabilities for every output state will change according to our experimental setup, while the number of calculated probabilities is still $\binom{10}{5}$=252. Intuitively, this architecture also allows to simulate photon losses, as they will be found in the additional modes m_{loss}. Each of these modes can be occupied by exactly one or zero photons.

The simulation is publicly available on github (*36*). It allows to determine simulated distribution of events for the validation protocols, as described in Materials and methods.

3 Time-bin Hong-Ou-Mandel experiment

In this section we discuss the results of the Hong-Ou-Mandel experiment performed in a time-bin fashion.

As mentioned in the main text, the measured value of the two-photon interference visibility $V^{(2)}$=(85.97±0.06)$\%$ is influenced by several factors. Here, we provide a comprehensive list of these factors, and discuss potential strategies for improving the $V^{(2)}$ values, in consequence improving the overall performance of the implementation.

Residual multi-photon emission. Quantum dot (QD) single-photon sources exhibit residual multi-photon emission, denoted by the second-order correlation function $g^{(2)}(0) > 0$. This arises from imperfections in laser filtering or re-excitation processes. Typically, correction factors are applied to account for the imperfect $g^{(2)}(0)$, allowing to extract an "intrinsic" single-photon indistinguishability (*43*) that is higher than the measured value by a factor $\sim 2g^{(2)}(0)$. In our case, this would result in an increase of the HOM visibilty of $\sim 3\%$. However, our time-bin HOM experiment incorporates an active element, that makes comparisons with pure passive optics configurations questionable. Consequently, we report the measured $V^{(2)}$ value rather than applying correction factors from the literature for imperfect $g^{(2)}(0)$.

Imperfect active switching and imperfect modulation of the laser pump beam. Both of these factors stem from equipment imperfections and can be reduced by employing improved devices (e.g., a higher-quality pulse generator to drive the fiber-based EOM modulating the pump laser). We can, however, estimate the contribution of these imperfections to the reduction in HOM visibility. In Fig. S4 we report the histogram of measured coincidences already shown in the main text, here including a zoomed-in view of correlation peaks at relevant time scales. The presence of a peak at Δt = 200 ns, which ideally should vanish, signifies the mentioned imperfections affecting all time-bins, including Δt = 100 ns. Subtracting the area under this peak from the areas under all the other peaks results in an increased HOM visibility of $V^{(2)} = (91.87 \pm 0.06)\%$.

Photon distinguishability at increasing time-scales. In our time-bin HOM experiment, we assess the indistinguishability of two photons in consecutive time-bins, specifically between the first and eighth consecutive photon of the stream emitted by the QD, separated by 100 ns. In this

scenario, a slight decrease in indistinguishability by a few percentage points can be anticipated as described in Ref. (*33*). In essence, photons emitted with longer temporal separations are slightly less indistinguishable from each other due to, e.g., spectral wandering of the quantum dot transitions. However, this effect is small in resonantly driven systems, like ours, hence we expect it to affect the levels of indistinguishability by only a few percentages.

4 Validation methods

Here, we briefly describe the methods we used to validate our experiment, that is, to give evidence that quantum interference between indistinguishable photons occurred in our interferometer. These are statistical tests which are widely employed for validating the Boson Sampling protocols on photonic platforms.

4.1 Aaronson-Arkhipov test

We first describe the validation test of Aaronson and Arkhipov against the hypothesis of uniform sampler (*40*). Let us indicate with U the $m \times m$ unitary matrix describing our m-mode interferometer, and with S = $\{s_1, ..., s_n\}$ the n modes occupied by the n input photons. Each single experimental outcome consists of n photons leaving the interferometer in a set of modes T = $\{t_1, ..., t_n\}$, as only the collision-free subspace is accessible in our experiment. Define a $n \times n$ submatrix $\overline{U}$ of U with elements $\overline{U}_{i,j} = U_{s_i,t_j}$. The method requires the calculation of the estimator $\mathcal{P} = \prod_{i=1}^{N} \sum_{i=1}^{N} |\overline{U}_{i,j}|^2$ where the index i labels the modes and the index j the output modes in which photons are detected. For the uniform distribution, the probability of one photon entering L in input i and exiting in output j is a constant (uniform) value $|\overline{U}_{i,j}|^2 = 1/m$ across any input/output setting, thus the estimator takes the form $\mathcal{P}^{unif.} = \left(\frac{n}{m}\right)^n$. If genuine boson interference occurs, one expects to observe more probable events more often. The method thus simply consists of defining a counter C_k initialized to $C_0 = 0$ and, for every detected event k, computing $\mathcal{P}$ and update the counter according to the following rule:

$$C_k := \begin{cases} C_{k-1} + 1 & \text{if} \quad \mathcal{P} \geq \left(\frac{n}{m}\right)^n \\ \\ C_{k-1} - 1 & \text{if} \quad \mathcal{P} < \left(\frac{n}{m}\right)^n, \end{cases} \tag{S1}$$

where n and m are the number of photons and modes in the interferometer.

A resulting positive counter then validates the quantum interference by rejecting the hypothesis that the data originates from the uniform sampler. Experimental evidence supporting the validity of the Aaronson-Arkhipov test was reported in Ref. (*44*).

4.2 Pattern Recognition Technique

This validation method, introduced in (*41*), works by the comparison of two samples, a *bona fide* one and a *test* o ne. Then, the goal is to state with which confidence level, the test sample belongs to the same probability distribution as the bona fide one.

Such a comparison is made through a coarse-graining of the data (i.e., a clustering) with an unsupervised machine learning method. This implies a clustering structure is built on the

bona fide sample, defining the distance between elements as the Euclidean one, on a given geometrical space. In particular, in the case of samples drawn from the input/output probabilities obtained through an experiment on a photonic circuit, the coordinates of this space are defined by the occupational numbers of the input/output combinations. For instance, in the case of an experiment with 5 photons injected in a $10-$mode circuit, each element will be represented by a $10-$ dimensional vector.

After the cluster structure is built, the elements of the test sample are put into it, by assigning each element to the cluster whose centroid (i.e., geometrical centre) is the closest. In the end, we compare the number of elements in each cluster belonging to the bona fide and the test sample and evaluate a χ^2 variable.

Since we are comparing two samples, the χ^2 variable is defined as follows:

$$\chi^2 = \sum_{j=1}^{N_s} \sum_{i=1}^{N_c} \frac{(N_{ij} - E_{ij})^2}{E_{ij}} \tag{S2}$$

where N_s is the number of samples that we are comparing (in our case $N_s = 2$), N_c is the number of clusters and N_{ij} is the number of elements of the j-th sample belonging to the i-th cluster. Then, $E_{ij} = \frac{N_i N_j}{N_{TOT}}$, where N_i is the total number of elements in the $i-$th cluster, N_j is the total number of elements amounting to the $j-$th sample and $N_{TOT} = \sum_i N_i = \sum_j N_j$.

The variable in Eq. (S2) behaves as a χ^2 variable with $(N_c - 1)$ degrees of freedom. From this quantity, the p-value can be easily calculated from standard χ^2 tables. The null hypothesis of the two samples coming from the same probability distribution is rejected if the p-value is lower than a threshold, which typically is either to 0.01 or 0.05 (in our case we use the latter).

In our case, the adopted clustering technique is the *K-means* one, with *k++* initialization (*?, 45*). Since the clustering can end up in local minima, for each comparison, we build 10 different cluster structures on the bona fide sample, from random initial states, and then consider only the lowest χ^2 coming out from the compatibility test.

In order to choose the proper hyperparameters for the algorithm, namely the optimal sample size and number of clusters for each tuple of number of photons and modes (n, m), we proceed as follows: we numerically generate 1000 pairs of samples drawn from the theoretical distribution, given by indistinguishable photon inputs going through the adopted optical circuit (also accounting for losses, see Supplementary Material). For each pair of samples, one constitutes the *bona fide* sample and the other the *test* one. Then, we apply the validation method illustrated above and evaluate the χ^2 value for all of the pairs (see Eq. (S2)) and the p-value corresponding to the mean of the χ^2 variables.

In parallel, we draw 1000 test samples from the probability given by distinguishable photon inputs going through the same optical circuit. Also in this case, we evaluate the χ^2 coming from the comparison of such samples with the bona fide ones generated before, as well as the p-value corresponding to the mean of the χ^2 variables.

We then go through this process for different sizes of the samples and for different numbers of clusters and check the accuracy of the algorithm. As an example, see Fig. S5, where we consider the 7-photons experiment, with a sample size of 486 events and a varying number of clusters. There we see that 10 is the lowest value of N_c which allows to correctly discard the samples drawn from the distinguishable photon probability distribution and validate the ones drawn from the indistinguishable photon one. Let us note that, for the 5- and 6-photons experiments, the algorithm could effectively discriminate the two cases with a smaller sample size than the available experimental data (i.e. respectively 300 and 700 events). On the contrary, for the 8-photons experiment, the algorithm is not able to correctly discard the distinguishable

photon input case, for the same sample size of the experimental data. So for this case, we select the hyperparameters maximizing the difference between the p-values in the two cases. At this point, once the optimal hyperparameters are selected, we can compare the bona fide samples with the experimental ones.

Figures

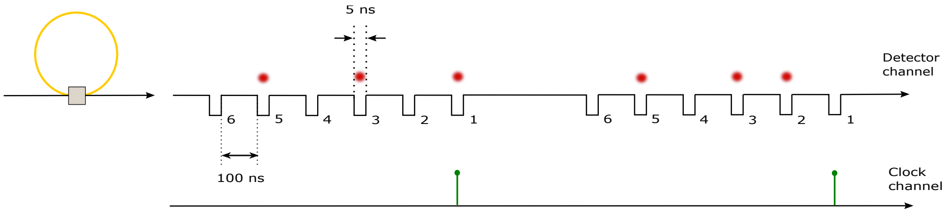

Figure S1: **Time-bin detection.** Schematics of the time-bin detection system for an experiment in which n=3 and m=6. The time-tagger registers on the ”detector channel” timestamps of single-photon detection events, represented by red circles. At the same time, the ”clock channel” registers the timestamps of an electrical square pulse signal, represented by green lollipop bars. The time distance between two clock timestamps defines the duration of a single experimental period. A time delay is introduced on all the detector channel timestamps, in order to synchronize the square pulses of the clock with the first time bin we want to examine. Then, for each experimental period, m time bins of 5 ns width are defined, equispaced in time by 100 ns. Once the time bins have been identified, a program loops over all the timestamps and for each clock pulse examines whether there are single-photon detection timestamps in the corresponding time bins and, if so, in which ones. In this example, we would measure two 3-photons events: the first one in the time bins (2,3,5) and the second one in the time bins (1,3,5).

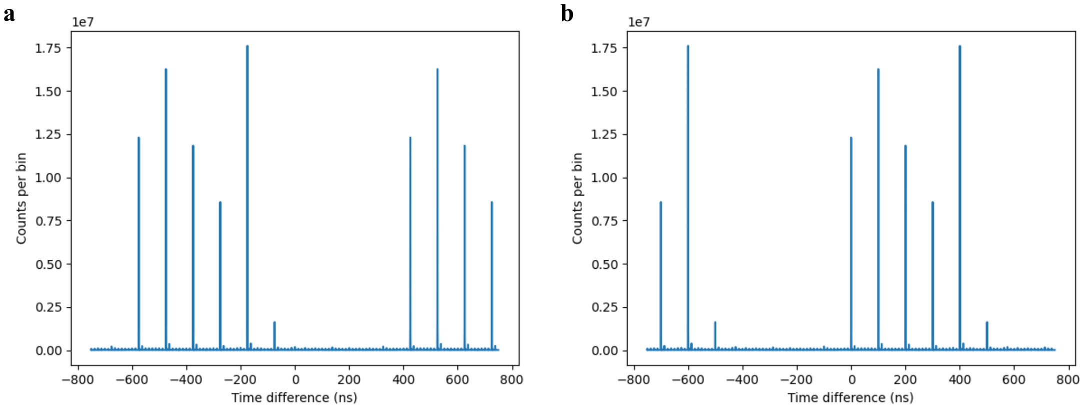

Figure S2: **Correlation histogram between the clock and the detector channels**. **a** Histogram of the raw data. The peaks identify the time bins in which the photons leave the interferometer. **b** The same histogram after introducing a time delay on all the timestamps registered on the clock channel, in such a way that they are synchronized with the first time bin of every experimental period.

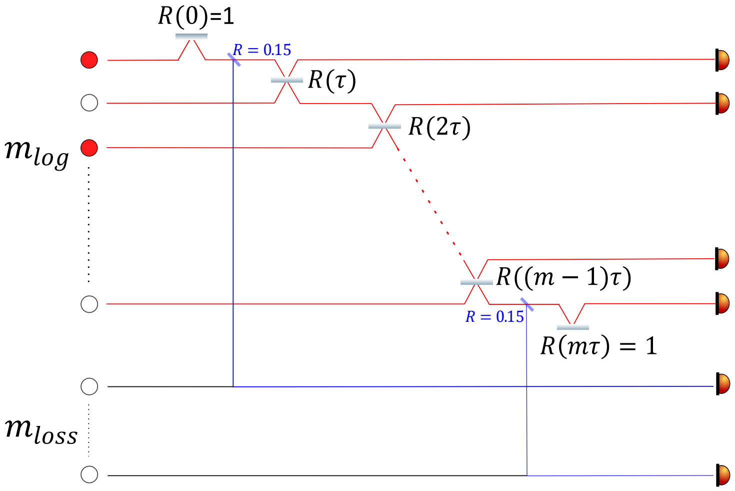

Figure S3: **Optical circuit with losses**. One extra loss channel is introduced to each logical channel by connecting them via a beamsplitter.

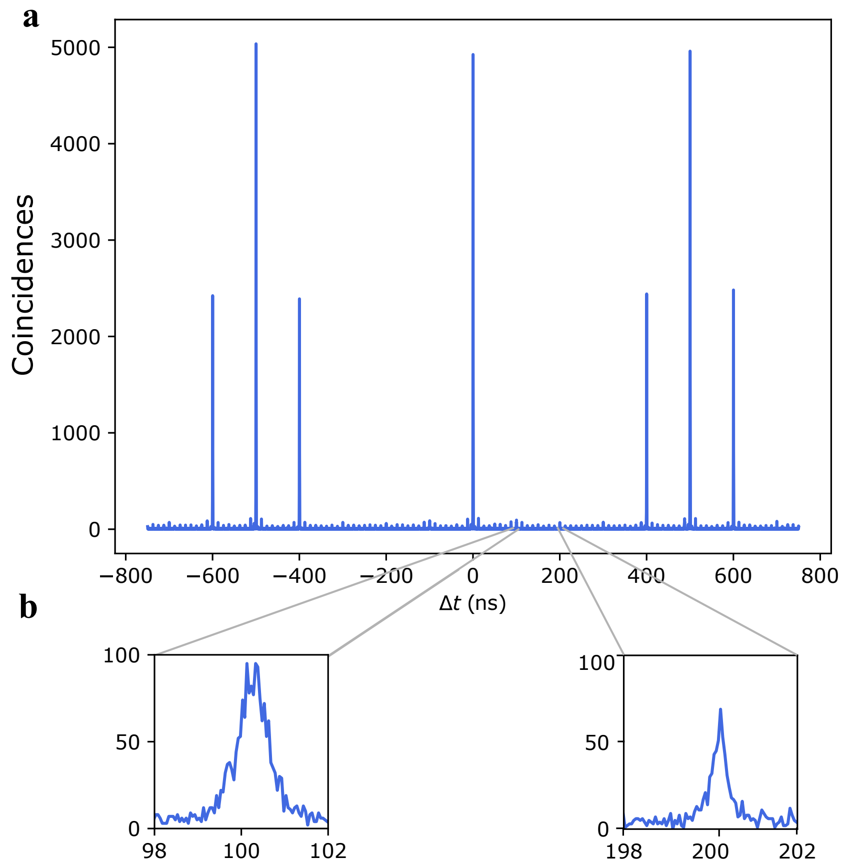

Figure S4: **Time-bin HOM intereference.** **a** A second-order autocorrelation measurement leads to correlations at Δt=0, for the bunching terms, and at Δt=100 ns, the time separation between consecutive time-bins. As expected for highly-indistinguishable photons, our data displays enhanced correlations at zero delay, and suppressed correlations at consecutive time-bins. **b** Zoom to the correlation peaks at relevant time-scales. The peak at Δt=200 ns—which should not exist in the ideal implementation—is an indicator of the imperfect active switching and imperfect modulation of the laser pump beam, affecting all time-bins, including Δt=100 ns.

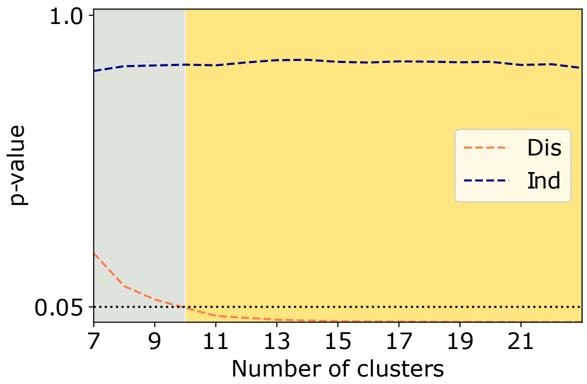

Figure S5: **Optimal hyperparameters choice (number of clusters).** We show the p-value coming from compatibility tests performed between *bona fide* samples and two different test samples. The bona fide samples are drawn from the theoretical distribution corresponding to 7 indistinguishable photons injected in a 14-mode optical circuit. Then, the first test sample is drawn from the same theoretical distribution ('Ind') and the second, from the one corresponding to a 7 distinguishable photon input ('Dis'). All of these samples contain 486 events. The red region indicates non-optimal choices for the number of clusters, as the algorithm is not rejecting the samples drawn from the distinguishable photon input probability distribution. Instead, in the green region, the algorithm performs correctly, as the p-value, in the second case, recognizes when two samples from different probability distributions are compared (i.e. it is below 0.05).

REFERENCES AND NOTES

1. F. Flamini, N. Spagnolo, F. Sciarrino, Photonic quantum information processing: A review. *Rep. Prog. Phys.* **82**, 016001 (2018).

2. S. Slussarenko, G. J. Pryde, Photonic quantum information processing: A concise review. *Appl. Phys. Rev.* **6**, 041303 (2019).

3. H. Wang, H. Yu, Y.-H. Li, S. Zu-En, B. Li, H.-L. Huang, X. Ding, M.-C. Chen, C. Liu, J. Qin, J.-P. Li, Y.-M. He, C. Schneider, M. Kamp, C.-Z. Peng, S. Höfling, C.-Y. Lu, J.-W. Pan, High-efficiency multiphoton boson sampling. *Nat. Photonics* **11**, 361–365 (2017).

4. J. Wang, S. Paesani, Y. Ding, R. Santagati, P. Skrzypczyk, A. Salavrakos, J. Tura, R. Augusiak, L. Mančinska, D. Bacco, D. Bonneau, J. W. Silverstone, Q. Gong, A. Acín, K. Rottwitt, L. K. Oxenløwe, J. L. O'Brien, A. Laing, M. G. Thompson, Multidimensional quantum entanglement with large-scale integrated optics. *Science* **360**, 285–291 (2018).

5. C. Taballione, R. van der Meer, H. J. Snijders, P. Hooijschuur, J. P. Epping, M. de Goede, B. Kassenberg, P. Venderbosch, C. Toebes, H. van den Vlekkert, P. W. H. Pinkse, J. J. Renema, A universal fully reconfigurable 12-mode quantum photonic processor. *Mater. Quantum Technol.* **1**, 035002 (2021).

6. H.-S. Zhong, Y. Li, W. Li, L.-C. Peng, S. Zu-En, H. Yi, Y.-M. He, X. Ding, W. Zhang, L. Hao Li, Z. W. Zhang, L. You, X.-L. Wang, X. Jiang, L. Li, Y.-A. Chen, N.-L. Liu, L. Chao-Yang, J.-W. Pan, 12-photon entanglement and scalable scattershot boson sampling with optimal entangled-photon pairs from parametric down-conversion. *Phys. Rev. Lett.* **121**, 250505 (2018).

7. H. Wang, J. Qin, X. Ding, M.-C. Chen, S. Chen, X. You, Y.-M. He, L. Xiao Jiang, Z. You, C. S. Wang, J. J. Renema, S. Höfling, L. Chao-Yang, J.-W. Pan, Boson sampling with 20 input photons and a 60-mode interferometer in a 10^{14}-dimensional hilbert space. *Phys. Rev. Lett.* **123**, 250503 (2019).

8. H.-S. Zhong, H. Wang, Y.-H. Deng, M.-C. Chen, L.-C. Peng, Y.-H. Luo, J. Qin, W. Dian, X. Ding, H. Yi, H. Peng, X.-Y. Yang, W.-J. Zhang, H. Li, Y. Li, X. Jiang, L. Gan, G. Yang, L. You, Z. Wang, L. Li, N.-L. Liu, L. Chao-Yang, J.-W. Pan, Quantum computational advantage using photons. *Science* **370**, 1460–1463 (2020).

9. J. M. Arrazola, V. Bergholm, K. Brádler, T. R. Bromley, M. J. Collins, I. Dhand, A. Fumagalli, T. Gerrits, A. Goussev, L. G. Helt, J. Hundal, T. Isacsson, R. B. Israel, J. Izaac, S. Jahangiri, R. Janik, N. Killoran, S. P. Kumar, J. Lavoie, A. E. Lita, D. H. Mahler, M. Menotti, B. Morrison, S. W. Nam, L. Neuhaus, H. Y. Qi, N. Quesada, A. Repingon, K. K. Sabapathy, M. Schuld, D. Su, J. Swinarton, A. Száva, K. Tan, P. Tan, V. D. Vaidya, Z. Vernon, Z. Zabaneh, Y. Zhang, Quantum circuits with many photons on a programmable nanophotonic chip. *Nature* **591**, 54–60 (2021).

10. H.-S. Zhong, Y.-H. Deng, J. Qin, H. Wang, M.-C. Chen, L.-C. Peng, Y.-H. Luo, W. Dian, S.-Q. Gong, S. Hao, H. Yi, H. Peng, X.-Y. Yang, W.-J. Zhang, H. Li, Y. Li, X. Jiang, L. Gan, G. Yang, L. You, Z. Wang, L. Li, N.-L. Liu, J. J. Renema, L. Chao-Yang, J.-W. Pan, Phase-programmable gaussian boson sampling using stimulated squeezed light. *Phys. Rev. Lett.* **127**, 180502 (2021).

11. J. B. Spring, P. L. Mennea, B. J. Metcalf, P. C. Humphreys, J. C. Gates, H. L. Rogers, C. Söller, B. J. Smith, W. S. Kolthammer, P. G. R. Smith, I. A. Walmsley, Chip-based array of near-identical, pure, heralded single-photon sources. *Optica* **4**, 90–96 (2017).

12. F. Graffitti, P. Barrow, M. Proietti, D. Kundys, A. Fedrizzi, Independent high-purity photons created in domain-engineered crystals. *Optica* **5**, 514–517 (2018).

13. Y. Xing Ding, Z.-C. D. He, M.-C. N. Gregersen, S. Chen, S. M. Unsleber, C. Schneider, M. Kamp, S. Höfling, L. Chao-Yang, J.-W. Pan, On-demand single photons with high extraction efficiency and near-unity indistinguishability from a resonantly driven quantum dot in a micropillar. *Phys. Rev. Lett.* **116**, 020401 (2016).

14. P. Senellart, G. Solomon, A. White, High-performance semiconductor quantum-dot single-photon sources. *Nat. Nanotechnol.* **12**:1026–1039 (2017).

15. R. Uppu, F. T. Pedersen, Y. Wang, C. T. Olesen, C. Papon, X. Zhou, L. Midolo, S. Scholz, A. D. Wieck, A. Ludwig, P. Lodahl, Scalable integrated single-photon source. *Sci. Adv.* **6**, eabc8268 (2020).

16. N. Tomm, A. Javadi, N. O. Antoniadis, D. Najer, M. C. Löbl, A. R. Korsch, R. Schott, S. R. Valentin, A. D. Wieck, A. Ludwig, R. J. Warburton, A bright and fast source of coherent single photons. *Nat. Nanotechnol.* **16**, 399–403 (2021).

17. F. Lenzini, B. Haylock, J. C. Loredo, R. A. Abrahão, N. A. Zakaria, S. Kasture, I. Sagnes, A. Lemaitre, H.-P. Phan, D. V. Dao, P. Senellart, M. P. Almeida, A. G. White, M. Lobino, Active demultiplexing of single photons from a solid-state source. *Laser Photonics Rev.* **11**, 1600297 (2017).

18. K. R. Motes, A. Gilchrist, J. P. Dowling, P. P. Rohde, Scalable boson sampling with time-bin encoding using a loop-based architecture. *Phys. Rev. Lett.* **113**, 120501 (2014).

19. A. Schreiber, A. Gábris, P. P. Rohde, K. Laiho, M. Stefaňák, V. Potoček, C. Hamilton, I. Jex, C. Silberhorn, A 2d quantum walk simulation of two-particle dynamics. *Science* **336**, 55–58 (2012).

20. D. Istrati, Y. Pilnyak, J. C. Loredo, C. Antón, N. Somaschi, P. Hilaire, H. Ollivier, M. Esmann, L. Cohen, L. Vidro, C. Millet, A. Lemaître, I. Sagnes, A. Harouri, L. Lanco, P. Senellart, H. S. Eisenberg, Sequential generation of linear cluster states from a single photon emitter. *Nat. Commun.* **11**, 5501 (2020).

21. X. Yu He, Z.-E. Ding, H.-L. Su, J. Huang, C. Qin, S. Wang, C. Unsleber, H. Chen, Y.-M. Wang, X.-L. He, W.-J. Wang, S.-J. Zhang, C. Chen, M. Schneider, L.-X. Kamp, Z. You, S. H. Wang, L. Chao-Yang, J.-W. Pan, Time-bin-encoded boson sampling with a single-photon device. *Phys. Rev. Lett.* **118**, 190501 (2017).

22. S. Sempere-Llagostera, R. B. Patel, I. A. Walmsley, W. S. Kolthammer, Experimentally finding dense subgraphs using a time-bin encoded gaussian boson sampling device. *Phys. Rev. X*, **12**, 031045, (2022).

23. Y. Enomoto, K. Yonezu, Y. Mitsuhashi, K. Takase, S. Takeda, Programmable and sequential gaussian gates in a loop-based single-mode photonic quantum processor *Sci. Adv* **7**, eabj6624 (2021).

24. K. Yonezu, Y. Enomoto, T. Yoshida, S. Takeda, Time-domain universal linear-optical operations for universal quantum information processing. *Phys. Rev. Lett.* **131**, 040601 (2023).

25. L. S. Madsen, F. Laudenbach, M. Falamarzi, F. R. Askarani, T. Vincent, J. F. F. Bulmer, F. M. Miatto, L. Neuhaus, L. G. Helt, M. J. Collins, A. E. Lita, T. Gerrits, S. W. Nam, V. D. Vaidya, M. Menotti, I. Dhand, Z. Vernon, N. Quesada, J. Lavoie, Quantum computational advantage with a programmable photonic processor. *Nature* **606**, 75–81 (2022).

26. M. Gimeno-Segovia, P. Shadbolt, D. E. Browne, T. Rudolph, From three-photon greenberger-horne-zeilinger states to ballistic universal quantum computation. *Phys. Rev. Lett.* **115**, 020502 (2015).

27. M. Lubasch, A. A. Valido, J. J. Renema, W. Steven Kolthammer, M. S. Dieter Jaksch, I. W. Kim, R. García-Patrón, Tensor network states in time-bin quantum optics. *Phys. Rev. A* **97**, 062304 (2018).

28. S. Takeda. A. Furusawa, Toward large-scale fault-tolerant universal photonic quantum computing. *APL Photonics* **4**, 060902 (2019).

29. P. P. Rohde, Simple scheme for universal linear-optics quantum computing with constant experimental complexity using fiber loops. *Phys. Rev. A* **91**, 012306 (2015).

30. Y. Li, Y.-H. Li, H.-B. Xie, Z.-P. Li, X. Jiang, W.-Q. Cai, J.-G. Ren, J. Yin, S.-K. Liao, C.-Z. Peng, High-speed robust polarization modulation for quantum key distribution. *Opt. Lett.* **44**, 5262–5265 (2019).

31. E. Meyer-Scott, N. Prasannan, I. Dhand, C. Eigner, V. Quiring, S. Barkhofen, B. Brecht, M. B. Plenio, C. Silberhorn, Scalable generation of multiphoton entangled states by active feed-forward and multiplexing. *Phys. Rev. Lett.* **129**, 150501 (2022).

32. C. K. Hong, Z. Y. Ou, L. Mandel, Measurement of subpicosecond time intervals between two photons by interference. *Phys. Rev. Lett.* **59**, 2044–2046 (1987).

33. J. C. Loredo, N. A. Zakaria, N. Somaschi, C. Anton, L. de Santis, V. Giesz, T. Grange, M. A. Broome, O. Gazzano, G. Coppola, I. Sagnes, A. Lemaitre, A. Auffeves, P. Senellart, M. P. Almeida, A. G. White, Scalable performance in solid-state single-photon sources. *Optica* **3**, 433–440 (2016).

34. Z.-C. Hui Wang, Y.-H. L. Duan, J.-P. Si Chen, Y.-M. Li, M.-C. C. He, X. Yu He, C.-Z. P. Ding, C. Schneider, M. Kamp, S. Höfling, L. Chao-Yang, J.-W. Pan, Near-transform-limited single photons from an efficient solid-state quantum emitter. *Phys. Rev. Lett.* **116**, 213601 (2016).

35. S. Becker, N. Datta, L. Lami, C. Rouzé, Convergence rates for the quantum central limit theorem. *Commun. Math. Phys.* **383**, 223–279 (2021).

36. T. Guggemos, "Python simulations for loop-based architecture"; https://github.com/CDL-Uni-Vienna/photonq-compiler/blob/main/LoopExperiment.ipynb.

37. N. Heurtel, A. Fyrillas, G. de Gliniasty, R. Le Bihan, S. Malherbe, M. Pailhas, E. Bertasi, B. Bourdoncle, P.-E. Emeriau, R. Mezher, L. Music, N. Belabas, B. Valiron, P. Senellart, S. Mansfield, J. Senellart, Perceval: A software platform for discrete variable photonic quantum computing. *Quantum* **7**, 931 (2023).

38. B. Seron, A. Restivo, Bosonsampling.jl: A julia package for quantum multi-photon interferometry. arXiv:2212.09537 [quant-ph] (19 December 2022).

39. D. J. Brod, E. F. Galvão, A. Crespi, R. Osellame, N. Spagnolo, F. Sciarrino, Photonic implementation of boson sampling: A review. *Adv. Photonics* **1**, 034001 (2019).

40. S. Aaronson, A. Arkhipov, Bosonsampling is far from uniform. arXiv:1309.7460 [quant-ph] (28 September 2013).

41. I. Agresti, N. Viggianiello, F. Flamini, N. Spagnolo, A. Crespi, R. Osellame, N. Wiebe, F. Sciarrino, Pattern recognition techniques for boson sampling validation. *Phys. Rev. X.* **9**, 011013 (2019).

42. S. Aaronson, D. J. Brod, Bosonsampling with lost photons. *Phys. Rev. A* **93**, 012335 (2016).

43. H. Ollivier, S. E. Thomas, S. C. Wein, I. M. de Buy Wenniger, N. Coste, J. C. Loredo, N. Somaschi, A. Harouri, A. Lemaitre, I. Sagnes, L. Lanco, C. Simon, C. Anton, O. Krebs, P. Senellart, Hong-Ou-Mandel interference with imperfect single photon sources. *Phys. Rev. Lett.* **126**, 063602 (2021).

44. N. Spagnolo, C. Vitelli, M. Bentivegna, D. J. Brod, A. Crespi, F. Flamini, S. Giacomini, G. Milani, R. Ramponi, P. Mataloni, R. Osellame, E. F. Galvao, F. Sciarrino, Experimental validation of photonic boson sampling. *Nat. Photon.* **8**, 615–620, (2014).

45. J. MacQueen, Some methods for classification and analysis of multivariate observations, in *Proceedings of the Fifth Berkeley Symposium on Mathematical Statistics and Probability* (University of California, Los Angeles, 1967), pp. 281–297.

46. D. Arthur, S. Vassilvitskii, K-means++: The advantages of careful seeding (Proceedings of the Annual ACM-SIAM Symposium on Discrete Algorithms, January 2007), vol. 8, pp. 1027–1035.
